# Supplementary material for: From sequence to enzyme mechanism using multi-label machine learning
Source: BMC Bioinformatics. 2014 May 19;15:150. doi: 10.1186/1471-2105-15-150 (PMC4229970; doi:10.1186/1471-2105-15-150)
Supplement: Additional file 2 — Java code of ml2db. Additional file ml2db_code.tar.gz contains the Java source code to run the multi-label machine learning experiments and save the results to database. The code’s Javadoc is included. [file 1471-2105-15-150-S2.zip › additional file 2/ml2db/utils/doc/index-files/index-16.html]

P-Index


JavaScript is disabled on your browser.


- Overview
- Package
- Class
- Use
- Tree
- Deprecated
- Index
- Help

- Prev Letter
- Next Letter

- Frames
- No Frames

- All Classes

A B C D E F G H I J K L M N O P Q R S T U V W X Y 


## P

Pareto - Class in cern.jet.random
:   Pareto Distribution.

Pareto(double, double, RandomElement) - Constructor for class cern.jet.random.Pareto
:   Constructs a Pareto distribution.

paretoFrequencies(Pareto, int) - Static method in class uk.ac.standrews.utils.main.stats.StatUtils
:   Extracts numbers from a pareto distribution for the given number of times
    and records the freqencies with which the integers have been extracted

ParetoTest - Class in uk.ac.standrews.utils.test.stats
:   Class

ParetoTest() - Constructor for class uk.ac.standrews.utils.test.stats.ParetoTest


parse(Reader) - Method in class uk.ac.standrews.utils.main.webutils.simpledomparser.SimpleDOMParser


parseAndGetSimpleElementTest() - Static method in class uk.ac.standrews.utils.test.SimpleDOMParserTest


PASSWORD\_PROP - Static variable in class uk.ac.standrews.utils.main.database.DbConn


PathUtils - Class in uk.ac.standrews.utils.main
:   Paths to utilities folders and files

PathUtils() - Constructor for class uk.ac.standrews.utils.main.PathUtils


pdf(double) - Method in class cern.jet.random.Pareto
:   Returns the probability distribution function.

PersistentObject - Class in cern.colt
:   This empty class is the common root for all persistent capable classes.

Pool - Class in uk.ac.standrews.utils.main.stringutils
:   Class

PORT\_PROP - Static variable in class uk.ac.standrews.utils.main.database.DbConn


post(URL, Map) - Static method in class uk.ac.standrews.utils.main.webutils.ClientHttpRequestUtils
:   posts a new request to specified URL, with parameters that are passed in
    the argument

post(URL, Map, Map) - Static method in class uk.ac.standrews.utils.main.webutils.ClientHttpRequestUtils
:   posts a new request to specified URL, with cookies and parameters that
    are passed in the argument

post(URL, Object[]) - Static method in class uk.ac.standrews.utils.main.webutils.ClientHttpRequestUtils
:   posts a new request to specified URL, with parameters that are passed in
    the argument

post(URL, String, Object) - Static method in class uk.ac.standrews.utils.main.webutils.ClientHttpRequestUtils
:   post the POST request specified URL, with the specified parameter

post(URL, String, Object, String, Object) - Static method in class uk.ac.standrews.utils.main.webutils.ClientHttpRequestUtils
:   post the POST request to specified URL, with the specified parameters

post(URL, String, Object, String, Object, String, Object) - Static method in class uk.ac.standrews.utils.main.webutils.ClientHttpRequestUtils
:   post the POST request to specified URL, with the specified parameters

post(URL, String, Object, String, Object, String, Object, String, Object) - Static method in class uk.ac.standrews.utils.main.webutils.ClientHttpRequestUtils
:   post the POST request to specified URL, with the specified parameters

post(URL, String[], Object[]) - Static method in class uk.ac.standrews.utils.main.webutils.ClientHttpRequestUtils
:   posts a new request to specified URL, with cookies and parameters that
    are passed in the argument

post() - Method in class uk.ac.standrews.utils.main.webutils.ClientHttpRequestUtils
:   posts the requests to the server, with all the cookies and parameters
    that were added

post(Map) - Method in class uk.ac.standrews.utils.main.webutils.ClientHttpRequestUtils
:   posts the requests to the server, with all the cookies and parameters
    that were added before (if any), and with parameters that are passed in
    the argument

post(Map, Map) - Method in class uk.ac.standrews.utils.main.webutils.ClientHttpRequestUtils
:   posts the requests to the server, with all the cookies and parameters
    that were added before (if any), and with cookies and parameters that are
    passed in the arguments

post(Object[]) - Method in class uk.ac.standrews.utils.main.webutils.ClientHttpRequestUtils
:   posts the requests to the server, with all the cookies and parameters
    that were added before (if any), and with parameters that are passed in
    the argument

post(String, Object) - Method in class uk.ac.standrews.utils.main.webutils.ClientHttpRequestUtils
:   post the POST request to the server, with the specified parameter

post(String, Object, String, Object) - Method in class uk.ac.standrews.utils.main.webutils.ClientHttpRequestUtils
:   post the POST request to the server, with the specified parameters

post(String, Object, String, Object, String, Object) - Method in class uk.ac.standrews.utils.main.webutils.ClientHttpRequestUtils
:   post the POST request to the server, with the specified parameters

post(String, Object, String, Object, String, Object, String, Object) - Method in class uk.ac.standrews.utils.main.webutils.ClientHttpRequestUtils
:   post the POST request to the server, with the specified parameters

post(String[], Object[]) - Method in class uk.ac.standrews.utils.main.webutils.ClientHttpRequestUtils
:   posts the requests to the server, with all the cookies and parameters
    that were added before (if any), and with cookies and parameters that are
    passed in the arguments

powlaw(double, double) - Method in class edu.cornell.lassp.houle.RngPack.RandomElement
:   generate a power-law distribution with exponent `alpha` and
    lower cutoff `cut`

printAllPaths(File) - Static method in class uk.ac.standrews.utils.main.FileUtils
:   Test method to print all absolute/relative paths of a file

printArray(Object[]) - Static method in class uk.ac.standrews.utils.main.collectionutils.ArrayUtils
:   Prints an array of objects

printArray(String[]) - Static method in class uk.ac.standrews.utils.main.collectionutils.ArrayUtils
:   Prints an array (String[])

printArrayOfArrays(double[][]) - Static method in class uk.ac.standrews.utils.main.collectionutils.ArrayUtils
:   Prints an array of arrays (String[][])

printArrayOfArrays(String[][]) - Static method in class uk.ac.standrews.utils.main.collectionutils.ArrayUtils
:   Prints an array of arrays (String[][])

printFromReader(BufferedReader) - Static method in class uk.ac.standrews.utils.main.FileUtils
:   Prints all lines from a reader.

printList(List) - Static method in class uk.ac.standrews.utils.main.collectionutils.ListUtils
:   Prints an array as a list of 'index' + 'array element' (with a header
    containing the size)

printListsOfLists(List<List>) - Static method in class uk.ac.standrews.utils.main.collectionutils.ListUtils
:   Prints lists of lists

printMap(Map) - Static method in class uk.ac.standrews.utils.main.maputils.MapUtils
:   Prints an HashMap as a list of index + key + value

printResultsSet(ResultSet) - Static method in class uk.ac.standrews.utils.main.database.DbUtils
:   Prints the results of a query

printTimestampedComment(String) - Static method in class uk.ac.standrews.utils.main.TimeUtils
:   Writes to System.out a timestamp + tab + the comment.

printTimestampedError(String) - Static method in class uk.ac.standrews.utils.main.TimeUtils


PropertiesUtils - Class in uk.ac.standrews.utils.main
:   Utilities to manipulate Java properties and properties text files

PropertiesUtils() - Constructor for class uk.ac.standrews.utils.main.PropertiesUtils


PseudoTruncatedPareto - Class in uk.ac.standrews.utils.main.stats
:   A truncated pareto distribution which stores a big lists of power-law
    extracted random numbers.

PseudoTruncatedPareto(double, double, int, int, int) - Constructor for class uk.ac.standrews.utils.main.stats.PseudoTruncatedPareto


PseudoTruncatedParetoSingleton - Class in uk.ac.standrews.utils.main.stats
:   Class

PseudoTruncatedParetoTest - Class in uk.ac.standrews.utils.test.stats
:   Class

PseudoTruncatedParetoTest() - Constructor for class uk.ac.standrews.utils.test.stats.PseudoTruncatedParetoTest

A B C D E F G H I J K L M N O P Q R S T U V W X Y

- Overview
- Package
- Class
- Use
- Tree
- Deprecated
- Index
- Help

- Prev Letter
- Next Letter

- Frames
- No Frames

- All Classes
